# Correlation between Mitochondrial Reactive Oxygen and Severity of Atherosclerosis

**DOI:** 10.1155/2016/7843685

**Published:** 2015-11-09

**Authors:** Gabriel G. Dorighello, Bruno A. Paim, Samara F. Kiihl, Mônica S. Ferreira, Rodrigo R. Catharino, Anibal E. Vercesi, Helena C. F. Oliveira

**Affiliations:** ^1^Department of Structural and Functional Biology, Biology Institute, State University of Campinas, 13083-862 Campinas, SP, Brazil; ^2^Department of Clinical Pathology, Faculty of Medical Sciences, State University of Campinas, 13083-887 Campinas, SP, Brazil; ^3^Department of Statistics, Institute of Mathematics, Statistic and Scientific Computation, State University of Campinas, 13083-859 Campinas, SP, Brazil

## Abstract

Atherosclerosis has been associated with mitochondria dysfunction and damage. Our group demonstrated previously that hypercholesterolemic mice present increased mitochondrial reactive oxygen (mtROS) generation in several tissues and low NADPH/NADP+ ratio. Here, we investigated whether spontaneous atherosclerosis in these mice could be modulated by treatments that replenish or spare mitochondrial NADPH, named citrate supplementation, cholesterol synthesis inhibition, or both treatments simultaneously. Robust statistical analyses in pooled group data were performed in order to explain the variation of atherosclerosis lesion areas as related to the classic atherosclerosis risk factors such as plasma lipids, obesity, and oxidative stress, including liver mtROS. Using three distinct statistical tools (univariate correlation, adjusted correlation, and multiple regression) with increasing levels of stringency, we identified a novel significant association and a model that reliably predicts the extent of atherosclerosis due to variations in mtROS. Thus, results show that atherosclerosis lesion area is positively and independently correlated with liver mtROS production rates. Based on these findings, we propose that modulation of mitochondrial redox state influences the atherosclerosis extent.

## 1. Introduction

Oxidative stress seems to be a common denominator unifying a variety of classic risk factors mode of action that leads to atherosclerosis [1]. The cellular sources of reactive oxygen species (ROS) are multiple, including NAD(P)H oxidase, xanthine oxidase, lipoxygenase, and cyclooxygenase systems, mitochondrial electron transport chain, and autoxidation of diverse substances. Previous studies found that ROS specifically derived from mitochondria (mtROS) are relevant in the context of atherosclerosis. For instance, mtROS induce endothelial dysfunction, infiltration and activation of inflammatory cells, and apoptosis of endothelial and vascular smooth muscle cells [2, 3]. In addition, a role for mitochondrial DNA damage and dysfunction in atherogenesis has been proposed in the last decade [4–9].

Our group have previously shown that mitochondria from various tissues of the hypercholesterolemic atherosclerosis-prone LDL receptor knockout mice (LDLr^−/−^) release more ROS than wild type controls [10]. In addition, this model presents reduced content of mitochondrial NADPH [10], the main reducing power for the antioxidant system glutathione and thioredoxine reductase/peroxidase [11]. This LDLr^−/−^ mitochondrial prooxidant state rendered the organelle more susceptible to membrane permeability transition (MPT). It has been well recognized that mitochondrial dysfunctions such as opening of the permeability transition pore directly promote inflammation and oxidative stress, phenomena involved in several cardiometabolic diseases [8, 9, 12]. The mitochondrial intrinsic pathway of cell death can be triggered by cholesterol accumulation in macrophages [13], an early event in atherogenesis. In addition, oxidative damage of mtDNA is correlated with human and mice severity of atherosclerosis [4, 9].

In a previous study, we verified that, besides NADPH deficiency, LDLr^−/−^ mitochondria contained less Krebs cycle intermediates such as isocitrate and that treating the organelle with isocitrate (*in vitro*) and the LDLr^−/−^ mouse model with citrate (*in vivo*) resulted in decreased mtROS production and increased NADPH content [14]. Major processes that consume cell NADPH and Krebs cycle intermediates are lipid and cholesterol biosynthesis, which are elevated in this animal model [10]. Therefore, we hypothesized that either citrate supplementation or cholesterol synthesis inhibition could increase mitochondria NADPH availability, decrease mtROS production, and decrease atherosclerosis development in LDLr^−/−^ mouse.

## 2. Material and Methods

### 2.1. Animals

Mating pairs of LDLr^−/−^ (B6.129S7-Ldlr<tm1Her./J, homozygous for Ldlr<tm1Her, stock number 002207) and wild type controls (C57BL6/J, stock number 000664) were purchased from The Jackson Laboratory (Bar Harbor, ME) in 2009 and both colonies have been established and maintained in strictly SPF conditions in The Multidisciplinary Center for Biological Investigation in the Area of Laboratory Animal Science (CEMIB) of the State University of Campinas (Unicamp). The CEMIB-Unicamp is part of the International Council for Laboratory Animal Science (ICLAS). The colonies are maintained through exclusive sibling mattings in order to maintain the homogeneous genetic background. When mice reach the age of 4 weeks, they are transferred to the Conventional Animal Facility in the Department of Structural and Functional Biology at Unicamp. Mice are then maintained in a temperature-controlled room (22 ± 1°C), with 12 h light/dark cycle, with 15 cycles of air changes per hour, and with free access to food (standard laboratory rodent chow diet, Nuvital CR1, Colombo, PR, Brazil) and filtered water. Four to five mice are maintained per cage. They are then enrolled in the experimental protocol which was approved by the Ethical Committee for the Use of Animal (CEUA/Unicamp, protocol # 1101-2) and by the Internal Biosecurity Committee (CIBio-IB/Unicamp, protocol # 2008/02). One-month-old male LDLr^−/−^ mice were randomly separated into four groups: control (CON), citrate (CIT), pravastatin (PRA), and citrate + pravastatin (CIT + PRA). The solutions of citrate (1.34 mM citric acid + 1.1 mM sodium citrate) and pravastatin (67 mg/L) equivalent to 10 mg/Kg of body weight were offered as the only source of drinking water. The solutions were provided in dark bottles and changed every other day during 3 months. Additional experiments were performed in untreated LDLr^−/−^ and wild type controls for checking differences in oxidative susceptibility status and oxidative lipid damage in their plasma VLDL particles, as well as for determining anti-inflammatory systemic markers. Control of the colony phenotype is done by measuring fasting plasma cholesterol levels (LDLr^−/−^ > 200 mg/dL and WT < 80 mg/dL) and genotype by PCR in tail tip DNA samples according to The Jackson Laboratory protocol.

### 2.2. Plasma Biochemical Analysis

Blood samples were collected from either the retroorbital plexus or the tail tip of anesthetized and overnight fasted mice. Total cholesterol, triglycerides, and nonesterified fatty acids were measured in fresh plasma using standard commercial kits (Roche-Hitachi, Germany, and Wako, Germany). Glucose levels were measured using a hand-held glucometer (Accu-Chek Advantage, Roche Diagnostic, Switzerland). The plasma urea, creatinine, alkaline phosphatase (ALP), alanine aminotransferase (ALT), and aspartate transaminase (AST) were determined using an automated modular analyzer PP (Hitachi) with Roche reagents (Roche Diagnostic, Germany). The plasma cytokines tumoral necrosis factor alpha (TNF*α*), interleukin 2 (IL-2), interleukin 4 (IL-4), and Interferon gamma (IFN*γ*) were measured with ELISA commercial kit (eBioscience, San Diego, CA) according to the manufacturer's instructions. Plasma oxidized LDL antibodies were measured in plasma as described by Zaratin et al. [15]. Polystyrene 96-well plates were coated with human copper-oxidized LDL and incubated overnight at 4°C. Plates were then blocked with 1% gelatin in PBS for 48 h at room temperature. After washing with PBS, plasma samples (1 : 200) were added and incubated for 2 h at room temperature. After washing with PBS containing 0.2 mL/L Tween 20, plates were incubated with 50 *μ*L of peroxidase conjugated rabbit anti-mouse IgG antibody at room temperature for 1 h. Finally, 75 *μ*L peroxidase substrate solution was added, incubated for 15 min, and ended by 25 *μ*L of 2 M sulfuric acid. The optical density (OD) was then measured at 450 nm. The results are presented as the OD readings (arbitrary units). CuSO_4_-induced plasma thiobarbituric acid reactive substances (TBARS) concentrations were determined by first exposing plasma to 0.1 mM CuSO4 at 37°C for 5 hours. Then, 100 *μ*L of oxidized plasma was incubated with 200 *μ*L of 0.7% thiobarbituric acid in 0.05 M NaOH and 60 *μ*L of 50% trichloroacetic acid. Samples were incubated in boiling water for 30 minutes, followed by centrifugation at 664 g for 15 minutes. The standard curve was prepared using several dilutions of 0.05 mM 1,1,3,3-tetramethoxypropane. The optical densities of samples and standard curve were measured in a microplate reader at 532 nm.

### 2.3. Body Composition and Tissue Lipids Analyses

The epididymal adipose tissue and liver and spleen fresh masses were determined gravimetrically. Mice carcass composition was determined by weighing carcass before and after water drying and before and after lipid extraction of the dried carcass with petroleum ether as previously described by Salerno et al. [16]. Liver lipids were extracted using the Folch method [17]. The liver content of cholesterol and triglycerides was determined using colorimetric-enzymatic assays (Roche-Hitachi, Germany) after dissolving the lipid extracts in a triton-containing buffer (0.1 M potassium phosphate, pH 7.4, 0.05 M NaCl, 5 mM cholic acid, and 0.1% Triton X-100).

### 2.4. Isolation of Mouse Liver Mitochondria

Mitochondria were isolated by conventional differential centrifugation at 4°C as previously described [18]. The livers were homogenized in 250 mM sucrose, 1 mM EGTA, and 10 mM Hepes buffer (pH 7.2). The mitochondrial suspension was washed twice in the same medium containing 0.1 mM EGTA and the final pellet was resuspended in 250 mM sucrose to a final protein concentration of 80–100 mg/mL. The protein concentration was determined by a modified Biuret assay.

### 2.5. Reactive Oxygen Species Production (ROS)

ROS production by mitochondria was monitored using the membrane-permeable fluorescent dye 2′,7′-dichloro-dihydro-fluorescein diacetate (H_2_DCF-DA) according to García-Ruiz et al. [19]. Briefly, mitochondria (0.5 mg/mL) were added to HANKS medium, pH 7.2, containing 1 *μ*M H_2_DCF-DA and a 5 mM mixture of NAD-linked substrates (malate + glutamate + *α*-ketoglutarate + pyruvate), in a 1 mL cuvette with constant stirring at 37°C. The fluorescence signal was recorded at the excitation/emission wavelength pair of 488/525 nm using a fluorometer (Hitachi, model F4500). Calibration was performed with known concentrations of dichlorofluorescein (DCF), which is the product of H_2_-DCF oxidation.

### 2.6. NADPH Oxidation Rates

The redox state of pyridine nucleotides was measured as previously described [10] in the mitochondrial suspension (1 mg/mL) containing 100 *μ*M EGTA, 5 mM succinate, and 5 *μ*M rotenone by following the fluorescence signal in a Hitachi F-4010 spectrofluorometer using excitation and emission wavelengths of 366 and 450 nm, respectively, and a slit width of 5 nm. The extent of pyridine nucleotide oxidation was calculated as a function of the fluorescence increase induced by isocitrate addition. Internal calibration was done by the addition of known amounts of NADH.

### 2.7. Histological Analysis of Atherosclerosis

*In situ* perfused hearts were excised and embedded in Tissue-Tek OCT compound (Sakura, USA), frozen at −80°C, cut in 10 *μ*m sections along 480 *μ*m aorta length from the aortic valve leaflets, and stained by Oil Red O according to Paigen et al. [20]. The lipid-stained lesions were quantified as described by Rubin et al. [21] using the* ImageJ* (1.45 h) software.

### 2.8. VLDL Oxidation Susceptibility

Plasma VLDL fractions were obtained from 12 h fasted mice by ultracentrifugation. Five hundred *μ*L of plasma was added to the bottom of the ultracentrifuge tube and carefully overlaid with 300 *μ*L of saline solution (150 mM NaCl, 1 mM EDTA, and density of 1.006). The samples were centrifuged at 140,000 rpm (1 × 10^6^ g), 16°C during 50 min in a microultracentrifuge, Hitachi (model CS150GXL). Two hundred *μ*L from the top layer containing the VLDL fraction was collected. The amount of VLDL used in the oxidation assay was normalized by the triglyceride concentration (100 mg/mL). CuSO_4_-induced oxidation (40 *μ*M, 37°C) was measured by detecting the formation of conjugated dienes at 234 nm over time (Spectrophotometer Fusion Packard) according to a previous report [22].

### 2.9. Lipid Chemical Markers Identification

VLDL samples were submitted to a Bligh-Dyer extraction [23]. Lipid extracts were resuspended in 50 *μ*L of methanol : H_2_O milliQ (1 : 1) and 10 *μ*L of the latter was diluted in 990 *μ*L of methanol and 0.1% formic acid. Data acquisition was performed in an LTQ-XL Orbitrap Discovery Instrument (30,000 FWHM, Thermo Scientific, Bremen, Germany) in the positive ion mode and at the *m*/*z* range of 600–1000 for complex lipid identification. Mass and intensity values for each spectrum were included in the Principal Component Analysis (PCA), which was performed using Unscrambler v.9.7 CAMO Software (Trondheim, Norway). After the discrimination of PCA, potential chemical markers were selected for identification. For this, structural propositions were performed using high resolution as the main parameter. Mass accuracy was calculated and expressed in terms of ppm shifts, according to Machado et al. [24]. Error values were considered with the assistance of online database Lipid MAPS (University of California, San Diego, CA; http://www.lipidmaps.org/) for guiding the choice of potential lipid markers.

### 2.10. Statistical Data Analyses

The results are presented as the means ± SE. The comparisons between the groups were analyzed by one-way ANOVA with posttest of Tukey. For correlation analyses, we performed Spearman's univariate correlation test, partial (adjusted) correlation, and multiple linear regressions models, using, respectively, GraphPad Prism 5.0, SPSS Statistics 14.0, and R package 2.9. In the multiple linear regressions analyses, variable selection in each model was performed using the* regsubsets* tool available in the* leaps* package for R [25, 26], which identifies the exploratory variables that create the best-fitting linear regression models according to Bayesian Information Criterion (BIC). The level of significance was set at *P* < 0.05.

## 3. Results

In a pilot experiment, diet-induced atherosclerosis was evaluated in LDLr^−/−^ treated or not with 120 mM citrate drinking solution [14] during two weeks. The high citrate concentration used in this study caused a marked increase in plasma lipid levels, without changes in mtROS production, and resulted in 80% increase in aortic atherosclerosis lesion area in the citrate treated LDLr^−/−^ mice (data not shown). Therefore, for the next studies, we employed a 50-fold lower citrate dose (2.4 mM) during 12 weeks and determined the spontaneous (not diet-induced) atherosclerosis development. This protocol did not cause elevation of plasma lipid levels. For the cholesterol inhibition group (statins treatment), we chose a low dose of a hydrophilic statin (pravastatin 10 mg/Kg/day) because we had previously shown that high statin dose can directly damage mitochondria [27]. This dose is proven to be still effective to reduce atherosclerosis in LDLr^−/−^ mice without important plasma lipid changes [28].

**Figure 1:**
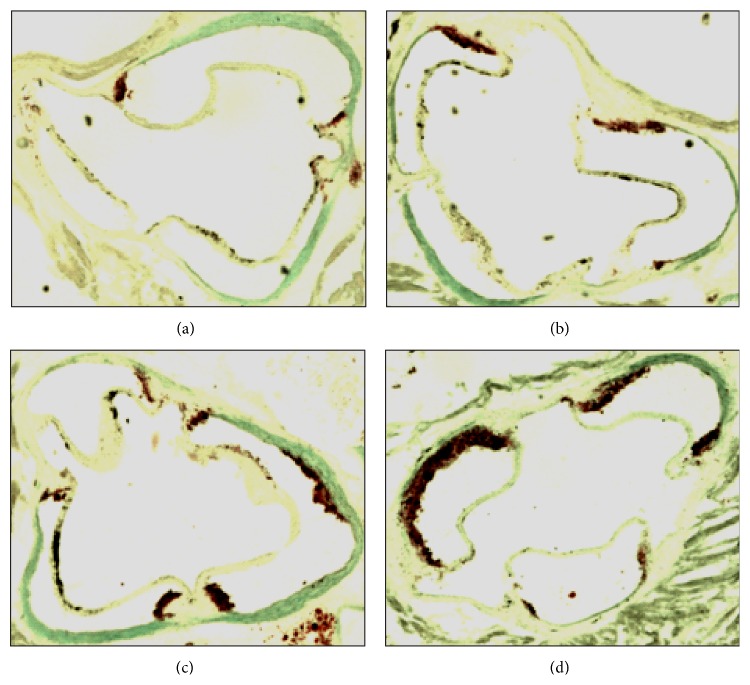
Representative images of aorta root with atherosclerosis lesions of LDL receptor knockout mice: control (a), treated with citrate (b), pravastatin (c), and citrate + pravastatin (d). The aortic root was cryosectioned and stained for lipids with Oil Red O and counterstained with light green.

Control (CON), citrate (CIT), pravastatin (PRA), and citrate + pravastatin (CIT + PRA) mice had similar body composition and plasma lipid levels (Table 1), although PRA group of mice presented lower body weight at the end of the treatment. The liver and renal functions were similar in all groups as verified through quantification of plasma alkaline phosphatase (ALP), alanine aminotransferase (ALT), aspartate transaminase (AST), and creatinine plasma levels. However, plasma urea levels in PRA group were 22% higher than in the other groups (Table 1). The average values of oxidative stress markers, such as susceptibility to lipid peroxidation (copper sulfate-induced thiobarbituric acid reactive substances), titles of plasma oxidized LDL antibodies, liver mitochondrial NADPH oxidation rates, and reactive oxygen species production rates (mtROS), were not significantly modified by the three treatments (Table 1). The development of spontaneous atherosclerosis, as measured by the area of lipid stained lesions in the aortic root, did not change significantly in CON, CIT, and PRA groups of mice. Surprisingly, the group CIT + PRA presented marked enhanced atherosclerosis (Table 1 and Figure 1).

**Figure 2:**
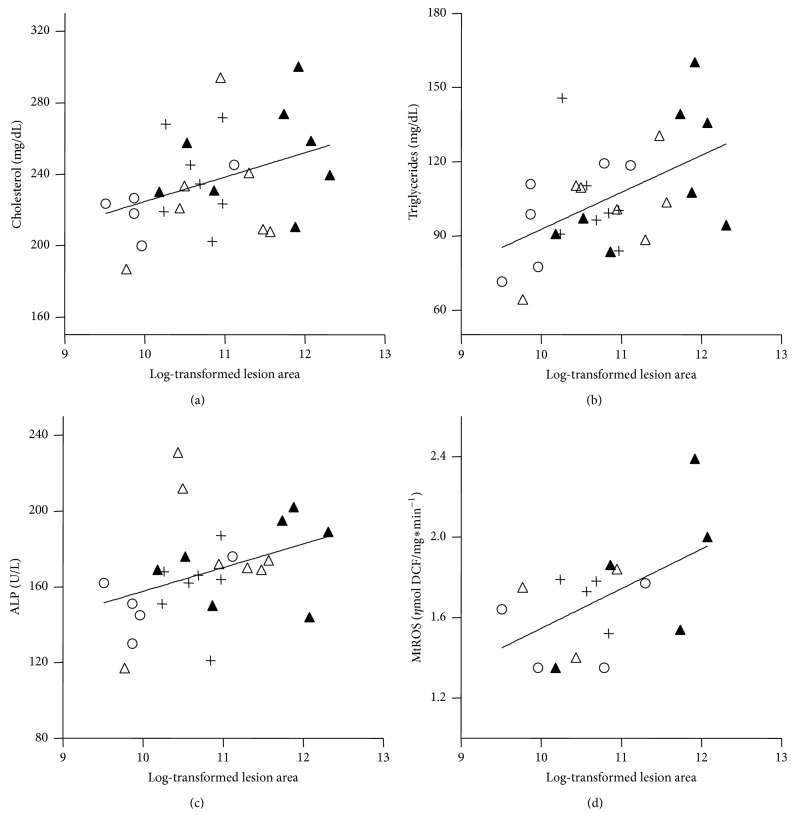
Spearman's correlations between log-transformed atherosclerosis lesion area (*μ*m^2^) and plasma cholesterol, *n* = 27 (a), plasma triglycerides, *n* = 28 (b), plasma alkaline phosphatase, *n* = 26 (ALP) (c), and liver mitochondria ROS production, *n* = 16 (d). ○ = control; + = citrate; △ = pravastatin; ▲ = citrate + pravastatin.

Since the treatments did not alter the average values of classic atherosclerosis risk factors such as plasma lipids, obesity, and oxidative stress, statistical correlation analyses in pooled group data were performed in an attempt to explain the differences observed in the variation of atherosclerosis lesion areas. Spearman's univariate correlation test showed that the atherosclerotic lesion areas were positively correlated with plasma cholesterol (*r* = 0.39, *P* = 0.046), triglycerides (*r* = 0.43, *P* = 0.026), ALP (*r* = 0.45, *P* = 0.02), and liver mitochondria ROS production (mtROS) (*r* = 0.54, *P* = 0.031) (Figure 2). We further tested the existence of linear multiple regression models to explain the extent of atherosclerosis. Three groups of variables were tested: (1) associations with body composition variables (carcass lean and fat mass, visceral fat mass, and body weight), (2) associations with plasma variables (glucose, triglycerides, cholesterol, free fatty acids, LDLox antibodies, and TBARS), and (3) associations with hepatic variables (liver mass, cholesterol and triglycerides content, and mtROS). For each model, variables that create the best-fitting linear regression are selected by the software [25, 26] to explain the atherosclerosis variation (Table 2). The body composition variables did not have any relevance in determining atherosclerosis variation (no significant associations were found). For the plasma variables, the software selected two significant models: (a) association with plasma triglyceride level and (b) association with plasma triglyceride and glucose level. For the hepatic variables, also two significant models were found: (a) association with mtROS and (b) association with mtROS and liver triglycerides content (Table 2). According to these regression models, the impact of these variables on the atherosclerosis extent can be interpreted as follows. Ten-unit increase in plasma triglycerides (10 mg/dL) is expected to increase lesion size by 17% (plasma model (2a)) and one unit increase in mtROS (nmol DCF/mg protein) is expected to increase the atherosclerotic lesion size by 370% (hepatic model (3a)).

In order to verify whether mtROS correlation with atherosclerosis was independent of the plasma triglyceride levels, we also performed the partial (adjusted) correlation test. This adjusted analysis included all variables that were significantly correlated with atherosclerosis by Spearman's correlation test (triglycerides, cholesterol, plasma ALP, and mtROS). After adjusting for plasma triglyceride levels, the only variable that remained significantly correlated with atherosclerosis was mtROS (*r* = 0.56, *P* = 0.047) (Table 2). Therefore, mtROS positive correlation with atherosclerosis is confirmed by three different statistical analyses and is independent of the variations of classical risk factors, the plasma lipid levels.

**Figure 3:**
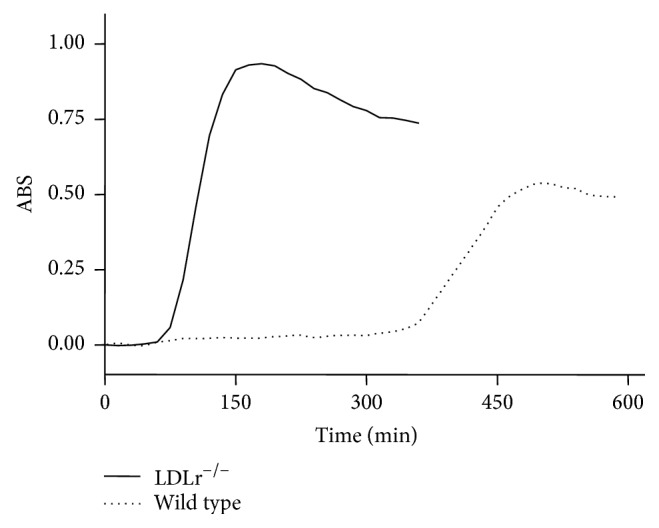
Representative curve of oxidation susceptibility of plasma VLDL obtained from LDL receptor knockout (LDLr^−/−^) and wild type mice. The VLDL oxidation was induced by incubation with CuSO_4_ (40 *μ*M). Formation of conjugated dienes was followed by absorbance changes at 234 nm along time. The VLDL lag-time to start oxidation was 73 ± 4.8 and 382 ± 79.9 minutes for LDLr^−/−^ and wild type mice, respectively (*P* = 0.0007). The VLDL oxidation rate, determined by the curve slopes, was about 2-fold greater (*P* < 0.0001) in the LDLr^−/−^ than wild type VLDL. The *n* used in this experiment was of 6–8 mice.

These statistical correlations between liver mtROS and atherosclerosis suggest a possible mechanistic link between liver and artery disease. The most likely connections are metabolic (for instance, secretion of oxidized VLDL) and/or an oxidative induction of proinflammatory cytokines. Therefore, we compared LDLr^−/−^ and wild type mice regarding VLDL susceptibility to oxidation (Figure 3) and lipid markers (Table 3) as well as the levels of plasma cytokines (Table 4). Figure 3 shows that VLDL secreted by LDLr^−/−^ livers is markedly more susceptible to an oxidative insult (CuSO_4_) than the wild type VLDL compared at the same TG content basis. Wild type VLDL shows a 5-fold greater lag time for oxidation initiation and a 50% lower rate of oxidation than VLDL from LDLr^−/−^ mice. In addition, mass spectrometry analyses of VLDL lipid extracts identified oxidized lipid markers, mainly phospholipids, in VLDL from LDLr^−/−^ compared with wild type mice (Table 3). Both groups were clearly separated with an accuracy of 95% and mass errors of less than 2 ppm. Regarding systemic inflammatory markers, Table 4 shows that three proinflammatory cytokines (TNF*α*, IL-2, and IFN*γ*) were augmented in the plasma of LDLr^−/−^ mice.

## 4. Discussion

Several studies have evidenced the relevance of mitochondria functionality to the development of atherosclerosis in humans and animals [2, 3, 7, 8]. Most studies agree that mitochondria ROS overproduction is likely implicated in atherogenesis and disease progression, although better mechanistic understanding is still needed [29].

Because we had previously found that mitochondria from atherosclerosis-prone mouse model (LDLr^−/−^) had decreased content of Krebs cycle intermediates, reducing equivalent power, and increased ROS release, we hypothesized that at least part of these mitochondrial features could be attributed to an elevated cholesterol synthesis, which consume both Krebs cycle intermediates and cell reducing equivalents. Therefore, in the present work, we tested whether citrate supplementation combined or not with inhibition of cholesterol synthesis could decrease mitochondrial ROS release and atherosclerosis development. In contrast to our hypothesis, any of the treatments affected significantly the average values of mtROS, and the combined citrate supplementation with pravastatin treatment actually increased atherogenesis.

Pravastatin alone increased plasma urea levels, and uremia* per se* appears to be proatherogenic [30]. Increased urea concentrations may increase the rate of isocyanate synthesis and increase carbamylation of lipids and proteins, including LDL [31]. Previous studies showed that carbamylated LDL dose dependently promotes proliferation of human coronary artery smooth muscle cells, endothelial cell death, and expression of adhesion molecules [32, 33]. On the other hand, in the group CIT + PRA, urea plasma levels were normal. It is possible that citrate treatment may have counteracted the effect of pravastatin on uremia, since it was previously shown that citrate reduced uremia induced by high fat diet [34]. Concerning atherosclerosis extent, it is difficult to explain why citrate + pravastatin would increase the progression of spontaneous atherosclerosis. We postulate that an interaction between these factors may have a local vascular wall harmful action, which we have not addressed in this study, but possibly related to mtROS production.

A limitation of this study is the low number (*n*) of mice and high variability of the responses. Therefore, we applied statistical methods to infer possible links between atherosclerosis and all measured parameters in pooled data. Correlation and regression analyses in pooled data help to identify which parameters related to risk factors (plasma lipids, obesity, and oxidative stress) have significant impact on the variation of atherosclerosis. As expected, plasma cholesterol and triglycerides were positively correlated to aortic lesion size, even within a very narrow range. Furthermore, new positive correlations were identified, named liver derived ALP and mtROS. Increasing levels of ALP suggest a correlation between liver damage and cardiovascular disease, which has already been shown by others regarding liver enzymes [35] and C-reactive protein levels [36]. However, after adjusting the analysis by plasma triglycerides, only mtROS remained positively and independently correlated with atherosclerosis. Multiple linear regression analysis also indicated that liver mtROS production could explain the extent of aortic atherosclerosis, either alone (Table 2, model (3a)) or in combination with liver triglycerides content (Table 2, model (3b)). Therefore, by using three distinct statistical tools (univariate correlation, adjusted correlation, and multiple regression) with increasing levels of stringency, we confirm a significant association between variables and further quantify the amount of variation in the dependent variable (atherosclerosis) that can be explained by the independent variable (mtROS). Although these statistical analyses do not imply causation, these results together with the whole body of evidences in the current literature lead us to propose that mechanistic links are conceivable. Liver mtROS could be linked to the arterial disease by different ways. The first one is through a metabolic connection, that is, by secreting altered amounts and/or quality of VLDL, the precursors of LDL. Secondly, liver mitochondrial oxidative stress results in activation of inflammatory pathways that may contribute to a systemic inflammation, which in turn is strongly correlated with atherosclerosis [36]. Finally, liver could be a surrogate marker of the main events occurring in the arterial wall, particularly lipid accumulation and mitochondrial oxidative stress. In support for the metabolic connection, we found that VLDL secreted by LDLr^−/−^ livers is markedly more susceptible to an oxidative insult (CuSO_4_) than the wild type VLDL, compared at the same TG content basis. In addition, these LDLr^−/−^ VLDL are already secreted with several oxidized lipids compared to the wild type VLDL. At this point, it is relevant to recall and quote Davis and Hui in their George Lyman Duff Memorial Lecture “Atherosclerosis is a liver disease of the heart” [37]. In that occasion, the authors referred specifically to the complex processes involved in the liver assembly and secretion of apoB-containing lipoproteins, the precursors of the atherosclerosis culprit, the LDL.

In conclusion, our results revealed that mitochondrial reactive oxygen is a novel positive and independent risk factor for atherosclerosis. We propose that modulation of mitochondrial redox state results in significant variation of atherosclerosis extent. These findings provide support for proposed strategies of using mitochondria-targeted antioxidants as potential therapy for treatment of cardiovascular diseases.

## Figures and Tables

**Table 1:** Body composition, plasma lipids and glucose levels, renal and hepatic function markers, systemic and mitochondrial redox parameters of LDL receptor knockout mice treated during 3 months with citrate and pravastatin.

Parameters	Control	Citrate	Pravastatin	Citrate + prava.
Body weight^1^	19.7 ± 0.2	17.7 ± 0.3	17.3 ± 0.4^*∗*^	18.2 ± 0.2
Carcass fat mass^2^	14.1 ± 0.7	15.6 ± 1.6	14.2 ± 1.0	13.1 ± 1.9
Carcass lean mass^2^	65.4 ± 0.9	64.5 ± 0.5	63.8 ± 0.6	64.9 ± 0.7
Visceral fat mass^3^	0.91 ± 0.08	0.93 ± 0.11	0.74 ± 0.07	0.84 ± 0.12
Liver mass^3^	4.68 ± 0.09	4.72 ± 0.08	4.63 ± 0.11	4.78 ± 0.04
Spleen mass^3^	0.27 ± 0.01	0.27 ± 0.01	0.25 ± 0.02	0.25 ± 0.00
Liver Cholesterol^4^	4.5 ± 0.6	5.1 ± 0.6	4.4 ± 0.6	5.9 ± 0.8
Liver Triglycerides^4^	79 ± 13.7	97 ± 17.8	69.3 ± 10.8	102.3 ± 15.6
Glucose^5^	85 ± 4.1	77 ± 4.1	85 ± 3.3	79 ± 4.2
Triglycerides^5^	103 ± 6.7	104 ± 6.6	110 ± 8.3	115 ± 8.8
Cholesterol^5^	234 ± 13.3	238 ± 8.4	238 ± 12.1	252 ± 9.1
Fatty acid^6^	0.80 ± 0.09	0.83 ± 0.06	0.75 ± 0.06	0.76 ± 0.03
LDLox antibodies^7^	0.34 ± 0.07	0.54 ± 0.08	0.42 ± 0.09	0.54 ± 0.07
TBARS^6^	258.5 ± 10.3	248.6 ± 7.8	234.5 ± 6.6	245.5 ± 15.2
ALT^8^	36.0 ± 2.1	48.7 ± 4.2	44.8 ± 4.5	38.7 ± 3.1
AST^8^	117.7 ± 11.4	138.1 ± 12.4	155.0 ± 27.9	130.0 ± 14.7
ALP^8^	158.1 ± 8	145.6 ± 15.7	173.1 ± 11.2	172.4 ± 7.7
Urea^5^	77.0 ± 2.4	84.7 ± 1.5	94.3 ± 5.4^*∗*^	81.8 ± 4.3
Creatinine^5^	0.12 ± 0.01	0.12 ± 0.01	0.12 ± 0.01	0.12 ± 0.01
mtROS production^9^	1.71 ± 0.19	2.02 ± 0.32	1.66 ± 0.08	1.83 ± 0.18
NADPH oxidation^10^	0.95 ± 0.13	0.80 ± 0.2	0.92 ± 0.21	0.73 ± 0.22
Atherosclerotic lesion area^11^	31.6 ± 8.8	43.8 ± 4.8	61.1 ± 12.8	116.8 ± 25.0^*∗*^

Data are mean ± SE (*n* = 8–10/group). Citrate (1.34 mM citric acid + 1.1 mM sodium citrate in the drinking water). Pravastatin (10 mg/Kg of body weight) in the drinking water (67 mg/L). ^1^g, ^2^% related to the dry carcass, ^3^% related to the body weight, ^4^mg/g of liver, ^5^mg/dL, ^6^ *μ*M, ^7^absorbance,  ^8^U/L, ^9^nM DCF/mg protein min^−1^, ^10^ *η*M NADPH/mg protein min^−1^, ^11^ *μ*m^2^ × 10^3^. ^*∗*^ *P* < 0.05.

**Table 2:** Multiple linear regression models for atherosclerosis and partial correlation adjusted by plasma triglycerides.

Statistical analyses	Significant parameters	Correlation coefficients	*P* value
(1) Body composition model^1^	None	0.06	0.12

(2) Plasma model^2^	(2a) Triglycerides	0.22	0.006
	(2b) Triglycerides and glycemia	0.25	0.01

(3) Liver model^3^	(3a) mtROS	0.23	0.035
	(3b) mtROS and liver triglycerides	0.28	0.047

Partial correlation adjusted by plasma triglycerides^4^	mtROS	0.56	0.047

Parameters tested in each model: ^1^carcass fat and lean mass, visceral fat mass, and body weight; ^2^plasma glucose, triglycerides, cholesterol, free fatty acids, LDLox antibodies, and TBARS; ^3^liver mass, hepatic cholesterol and triglyceride contents, and liver mitochondria ROS production; ^4^plasma cholesterol, plasma alkaline phosphatase, and liver mitochondria ROS production were tested after adjustment by plasma triglycerides.

**Table 3:** Lipid chemical markers identified by high resolution electrospray ionization mass spectrometry (ESI-MS) analysis of plasma VLDL from LDL receptor knockout and wild type mice.

	Molecule	Theoretical mass	Experimental mass	Error (ppm)	LM ID^*∗*^
Wild type	[PE(15:1/22:4)+K]^+^	790,4784	790,4769	−1,8976	LMGP02010503
	[TG(14:0/16:1/20:1)+K]^+^	869,6995	869,7010	1,7247	LMGL03014259
	[PG(18:0/13:0)+Na]^+^	715,4884	715,4889	0,6988	LMGP04030029
	[PC(13:0/18:2)+H]^+^	716,5225	716,5238	1,8143	LMGP01011348
	[PC(13:0/18:4)+Na]^+^ + OH	751,4764	751,4763	−0,1331	LMGP01011351
	[PI(12:0/20:5)+Na]^+^	823,4368	823,4379	1,3359	LMGP06010031
	[PE-Cer(15:2/20:0)+Na]^+^ + OH	711,5048	711,5059	1,5460	LMSP03020077
	[PE-Cer(14:1/22:1)+K]^+^	725,4994	725,5002	1,1027	LMSP03020009
	[PE-Cer(15:1/20:0)+K]^+^ + 2OH	729,4943	729,4934	−1,2337	LMSP03020074
	[PA(20:4/20:0)+K]^+^	791,4988	791,4975	−1,6425	LMGP10010636

LDLr^−/−^	[PC(16:1/18:1)+H]^+^ + OH	759,5778	759,5791	1,7115	LMGP01090011
	[PC(19:0/15:1)+H]^+^	760,5851	760,5839	−1,5777	LMGP01011732
	[PG(19:1/18:2)+H]^+^	787,5484	787,5472	−1,5237	LMGP04010493
	[PS(18:0/16:0)+H]^+^ + OH	781,5469	781,5477	1,0236	LMGP03010888
	[PC(18:3/18:1)+H]^+^ + OH	783,5778	783,5765	−1,6591	LMGP01090030
	[PC(20:5/18:1)+H]^+^ + OH	807,5778	807,5766	−1,4859	LMGP01090051
	[PA(22:2/16:0)+K]^+^ + OH	785,5093	785,5103	1,2731	LMGP10010761
	[PS(16:0/17:2)+H]^+^ + 2OH	762,4916	762,4901	−1,9672	LMGP03030011
	[PS(16:0/20:5)+H]^+^ + OH	782,4967	782,4976	1,1502	LMGP03030022
	[PI(14:0/22:6)+Na]^+^	877,4838	877,4845	0,7977	LMGP06010892

PE: phosphatidylethanolamine; TG: triacylglycerols; PG, phosphoglycerol; PC: phosphatidylcholine; PI: phosphatidylinositol; PS: phosphatidylserine; Cer: ceramide; PA: phosphatic acid. ^*∗*^LM ID: Lipid MAPS identity. Identification is based on exact mass of each compound and Lipid MAPS database. The assigned IDs are for general structures and can be any of the positional isomers.

**Table 4:** Plasma cytokines of LDL receptor knockout and control wild type mice.

Cytokines (pg/mL)	LDLr^−/−^	Wild type	*P* value	*N*
TNF*α*	1364 ± 255	467 ± 53	0.007	8–10
IL-2	58.8 ± 10.7	28.1 ± 2.7	0.02	7-8
IFN*γ*	1553 ± 238	819 ± 124	ns	8–10

Data are mean ± SE. *P* value from Student's *t*-test. ns: nonsignificant.
